# Transcriptome Analysis of the SL221 Cells at the Early Stage during *Spodoptera litura* Nucleopolyhedrovirus Infection

**DOI:** 10.1371/journal.pone.0147873

**Published:** 2016-02-03

**Authors:** Qian Yu, Youhua Xiong, Jianliang Liu, Dongling Wen, Xiaohui Wu, Hanqi Yin

**Affiliations:** 1 College of Food Science and Technology, Zhongkai University of Agriculture and Engineering, Guangzhou, Guangdong Province, 510225, P. R. China; 2 School of Life Sciences, Sun Yat-Sen University, Guangzhou, Guangdong Province, 510275, P. R. China; 3 Shanghai Biotechnology Corporation, Shanghai, 201203, P. R. China; Chinese Academy of Fishery Sciences, CHINA

## Abstract

*Spodoptera litura (S*. *litura)* is one of the most destructive agricultural pests worldwide. There is urgent need for a nuclear polyhedrosis virus that is specific to *S*. *litura*. To date, there have been no reports regarding the responses of *S*. *litura* cells to early *Spodoptera litura* nucleopolyhedrovirus (SpltNPV) infection due to the lack of a reference genome and transcriptome for *S*. *litura*. In this study, a cell transcriptome from the host *S*. *litura* was assembled and used for Illumina strand-specific RNA sequencing (RNA-seq) to generate 99180 unigenes, representing the 18 hour infection cycle. More than 2000 *S*. *litura* genes were significant differentially regulated throughout the infection. The levels of viral mRNAs began to increase dramatically at 6 hpi, and this increase continued throughout the remainder of the infection. We focused on the expression of genes related to stress responses, apoptosis, metabolic enzymes and host cell innate immune system. A small subset of genes related to host stress response, especially for 62 ones being able to annotated as enzyme, ligand and receptor genes, were observed to be specifically differentially expressed at 6 hpi. At 18 hpi, 104 unigenes were continuously significantly changing from 0 hpi to 18 hpi, considered to be viral multiplication related genes, including 3 annotated SL221 unigenes and 81 viral genes, such as *tetraspanin* and *iap* gene. This information and further studies on the regulation of host gene expression by baculovirus infection at early stage will provide the tools needed to enhance the utility of this virus as an effective insecticide.

## Introduction

*Spodoptera litura (S*. *litura)*, previously known as a sporadic pest of various crops in Andhra Pradesh and India, has now assumed major pest status in many countries, including Japan, China and various Southeast Asian countries [1,2]. As one of the most destructive pests of agricultural crops worldwide, this highly polyphagous insect attacks over 80 species of agricultural crops and greening plants. This pest may become a serious problem during the seedling stage, and it has been reported that its resistance to certain insecticides is increasing [3,4]. Various different studies have shown that excessive reliance on chemical insecticides for *S*. *litura* control has lead to the development of multiple resistances, and the insecticides also eliminate the natural enemies of *S*. *litura* [5–8]. The development of integrated control programmes including the use of the baculoviruses that are specific to *S*. *litura*, is urgent.

Baculoviruses are viruses with circular double-stranded DNA genomes that replicate in the nuclei of infected host cells [9,10]. Baculoviruses replicate only in invertebrates, and many of them are highly pathogenic to important insect pest species [11]. Because of these advantages, baculoviruses have been used as biological control agents in agriculture and forestry, and there is great interest in further improving them for this purpose [12–14]. *Spodoptera litura* nucleopolyhedrovirus (SpltNPV) has been successfully applied as a large-scale commercial biological insecticide against the cotton leaf worm in China [15,16]. A total of 189 clones from uncloned SpltNPV stocks have been collected throughout Japan and these clones have been classified into three types: A, B and C [17–19]. However, SpltNPV genotype strain G2 isolated from ZSU strain [20], showing low proliferation efficiency (normal multiplicity of infection (MOI) less than 1) in host *S*. *litura* SL221 cells was puzzling. Generally, baculoviruses change host cells by arresting host cell cycle progression, crafting a “viral pseudo S phase”, then remodeling the cellular cytoskeleton, forming virogenic stroma in the nucleus as a specialized niche for replication, undertaking transcription and packaging, and eventually breaking-down the host nuclei [21]. We speculated that SL221 cells availably response to SpltNVP-G2 infection by against cell cycle arrest may be the rate controlling step for the following virus propagation. So in this study we focused on SpltNPV-G2 infected SL221 cells at gene level especially during the early stage of infection.

Thus far, considerable information regarding viral gene expression in infected host cells has been obtained. A rich knowledge about baculovirus gene expression profiles and gene functions during infection have been produced [10,22,23]. Although SpltNPV-G2 genome was completely sequenced [17], there have been no reports regarding the responses of the *S*. *litura* cell to SpltNPV infection due to the lack of a reference genome and transcriptome for *S*. *litura*. In this study, a cell transcriptome from the host *S*. *litura* SL221 cells was assembled and used to analyze changes in host cell gene expression throughout the early infection of SpltNPV using Illumina strand-specific RNA sequencing (RNA-seq). RNA-seq is a powerful tool for transcript discovery, genome annotation and expression profiling [24,25]. This study and future investigations of the regulation of host gene expression and the events of the SpltNPV infection cycle will provide new tools needed to enhance the utility of SpltNPV as an insecticide, and they will stimulate new approaches to understanding, modifying, and utilizing baculoviruses for various applications including protein expression.

## Materials and Methods

### Ethics Statement

All S.litura used in the present study were destructive pest and bred in State Key Laboratory of Biocontrol for research (Sun Yat-sen university, Guangzhou, China). No specific permits were required for S.litura sample collection or described sampling. The location was not privately-owned or protected, and the field studies did not involve any endangered or protected specie.

### Cell Line and Viruses

A single large RNA-seq dataset obtained from uninfected and SpltNPV-infected *S*. *litura* cell SL221 cells was used for extensive analysis of SpltNPV transcripts, the dynamics of the SpltNPV transcriptome, and the overall events of the cellular responses to SpltNPV infection. SL221 cells were cultured in TNM-FH medium (Invitrogen, Carlsbad, CA) supplemented with 10% FBS at 28°C as described previously, and they were infected with a wild-type SpltNPV genotype strain G2 [26]. Virus titers were determined by TCID50 (50% tissue culture infective dose) end-point dilution assay using SL221 cells. For infection experiments, SL221 cells were grown to the desired cell density, and one million SL221 cells were inoculated with SpltNPV (MOI = 1) in a T25 flask as described previously. After a 1-hour incubation, the inoculum was removed, and the cells were rinsed with Grace's medium and cultured in TNM-FH medium supplemented with 10% FBS at 28°C. The time at which the inoculum was removed was designated 0 h post infection (hpi). After incubation at 28°C for various lengths of time (0, 6, 12, 18 hpi), total RNA was isolated from SpltNPV-infected cells using a Qiagen RNeasy Mini kit according to the manufacturer’s instructions. Each time point was repeated for three times.

### Illumina Strand Specific RNA Sequencing

Illumina sequencing libraries were constructed following a modified strand-specific RNA sequencing protocol [27]. In brief, 3 μg purified total RNA was used for isolation of coding RNA and non-coding RNA using a ribo-zero kit for rRNA depletion, and isolated RNA samples were simultaneously eluted and fragmented in Elute-Prime-Fragment Mix at 94°C for 8 min. First-strand cDNA synthesis was carried out using SuperScriptII (Invitrogen, Carlsbad, CA) in the presence of random hexamer primers. Second-strand cDNA was synthesized at 16°C for 1 hour. After end-repair and dA-tailing, the DNA fragments were ligated with TruSeq adapters. The sample was amplified with TruSeq PCR primers and sequenced on the Illumina HiSeq2500 platform at Shanghai Biotechnology Corporation. (Note: 2 × 100 paired end sequencing for one of the group samples were used for unigene assemble and differential expression analysis as described below. The other 1 × 50 single end sequencing groups were only used for differential expression analysis.)

### SL221 Transcriptome Assembly and Unigene Annotation

To filter out low-quality nucleotides, adapters, and PCR primer sequences, the raw RNA-seq reads were processed with the ShortRead package. Reads with a length between 35 nt and with at least 2 N (ambiguous nucleotides) were removed. In addition, reads that mapped to the SLIVA database (http://www.arb-silva.de/download/arb-files/) were discarded. The resulting cleaned reads of 2 × 100 paired end sequencing were then assembled as unigenes using the Trinity package with an optimized k-mer length of 25 [28,29]. The assembled unigenes of all the samples were cleaned by removing redundancies and were further assembled as all-unigenes using CD-HIT software [30]. The cleaned reads were strand-specifically aligned to the assembled all-unigenes using FANSe2 allowing 7 nt of mismatch [31,32]. The all-unigenes with at least 10 mapped reads were considered reliably assembled unigenes.

The COG and KEGG pathway annotations were performed via blastx search against the UniProt (UniProtKB/TrEMBL), Kyoto Encyclopedia of Genes and Genomes database (KEGG) and Cluster of Orthologous Groups (COG) databases with an E-value threshold of 1e-05. Gene ontology (GO) analysis is a functional analysis that associates differentially expressed genes with GO categories. For each annotated sequence, successful blast hits were strand-specifically mapped, and Blast2GO was used to annotate the entire set of unigenes. GO functional classification for unigenes was performed using WEGO [33,34].

The directions and CDS (coding sequences) of the unigenes that were not aligned to sequences in any of the databases mentioned above were analyzed using Trinity transdecoder software. Coding-Non-Coding Index (CNCI) software was used to predict long noncoding RNAs (lncRNAs) within the unigenes without the annotations or CDS sequences mentioned above, with a cutoff score < -0.1.

### Identification of Differentially Expressed Unigenes

The reads from 1 × 50 single end sequencing samples were strand-specifically aligned to the assembled unigenes using FANSe2 allowing 3 nt of mismatch [31,32]. To analyze unigenes that were differentially expressed, the counts of each unigene in each sample was applied trimmed mean of M-values (TMM) normalization and Estimating the common dispersion (estimateCommonDisp) using the edgeR package [35,36]. Moderated log-CPM (counts per million) was used to calculate the expression levels of differentially expressed unigenes at different times post infection, by comparing data from virus-infected cells to infected cells at the adjacent time point or at 0 h. Genes with a P-value lower than 0.05 and fold change over |2| were categorized as differentially expressed. For the identification of significantly enriched GO terms, all of the identified differentially expressed genes (DEGs) were mapped to GO enrichment analysis of functional significance. In the pathway enrichment analysis, all DEGs were mapped to terms in the KEGG database.

### Validation of Gene Expression

Four genes identified in this transcriptome sequencing analysis were selected for confirmation by real-time PCR (RT-qPCR) using 2× SYBR green real-time PCR mix (Takara, Madison, WI)according to the manufacturer's protocol, and GAPDH were used as an endogenous control. The prepared total RNA used in RT-PCR analysis was isolated from three biological replicate samples as that used for RNA-seq. The RT-qPCR was performed on the ABI 7500 real-time PCR system (ABI, USA). The primers used for qPCR of selected genes were designed according to RNA-seq data with Primer Premier 5 (PREMIER Biosoft international, Palo Alto, CA, USA). The PCR amplification was performed in triplicate, using the following cycling parameters: 94°C for 2 min, followed by 40 cycles of 15 s at 94°C, 15 s at 60°C, and 34 s at 72°C. All samples were analyzed in triplicate and the relative target gene expression (means ± SD) was calculated as relative fold changes using the 2^−ΔΔCT^ method. Statistical analyses were conducted using using Student’s t test. Differences were considered statistically significant at P < 0.05.

## Results

### Assembly and Annotation of the *S*. *litura* SL221Cell Transcriptome

To analyze the cellular responses to SpltNPV infection, the *S*. *litura* cell line SL221 was infected with wild-type baculovirus SpltNPV at a MOI of 1. RNAs were isolated at three time points throughout the infection cycle (6 hpi, 12 hpi and 18 hpi) and were analyzed in comparison with 0 hpi (mock-infected) cells. After discarding ribosomal RNA and low-quality reads, 47,156,978, 42,959,338, 37,823,204 and 47,840,494 high-quality reads were obtained from mock-infected, 6 hpi, 12 hpi and 18 hpi cells for the 2 × 100 paired end sequenced samples, respectively. After subsequent assembly, 139,440, 135,137, 100,335 and 126,394 unigenes were obtained, respectively. All of the four sets of unigenes were further assembled as described in Materials and Methods. Finally, a total of 99,180 unigenes with at least 10 reads mapping to at least one sample were assembled with an N50 value of 1,798 nt and an average length of 1,096 nt (Table 1). A sequence similarity search was conducted by using BLAST. The search identified 23,630 unigenes (23.8%) that showed similarities to existing GenBank entries (S1 Table). These annotated unigenes showed significant similarity to sequences from insect species such as *Danaus plexippus* (7,911 members), *Bombyx mori* (7,753 members), *Pararge aegeria* (2527 members), *Acyrthosiphon pisum* (829 members), *Papilio xuthus* (410 members), and *Tribolium castaneum* (298 members) according to the annotations in UniProt Protein database (S1 Fig). In addition, 35 members, including 91 out of 141 ORFs in the SpltNPV genome with protein identifying greater than 80% (140 ORFs with E-value < 1e-5), were included in these unigene sequences. Therefore, the SpltNPV sequence was confirmed by *de novo* assembly of the genome from the viral transcriptome. Approximately 98% of the unigenes longer than 300 bp were annotated.

**Table 1:** Assembly statistics for the SL221 transcriptome.

N25	N50	N75	Longest	Mean	Median	shortest	N_unigenes	Annotated
3,898	1,798	745	21,889	1,096	599	201	99,180	23,630

### Gene Ontology, KEGG Pathway and COG Classification

**Fig 1:**
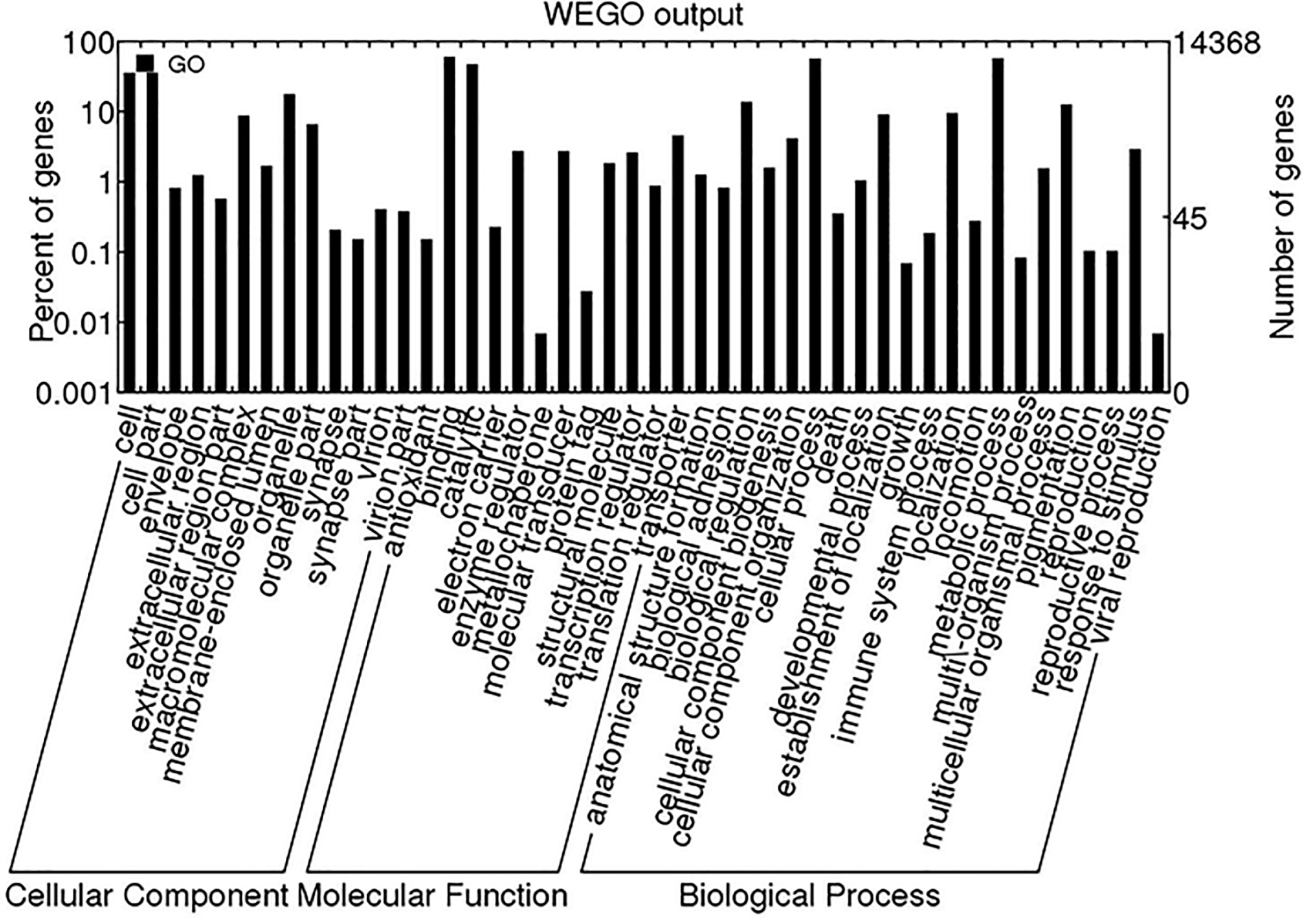
Gene ontology (GO) term distribution. A total of 17,196 unigenes were classified by WEGO software to perform the GO functional classification.

GO terms were assigned to the *S*. *litura* unigenes based on their sequence matches in the UniProt and Pfam domain databases. A total of 17,196 (17.3%) unigenes were assigned to at least one GO term, of which 13,139 (13.2%), 6,361 (6.4%) and 15,003 (15.1%) unigenes were assigned to GO terms in the categories of cellular components, molecular functions, and biological processes, respectively (Fig 1). In addition, a substantial number of unigenes (15,027, approximately 15.1%) were assigned to GO terms in two or more categories.

To identify the potential involvement of the annotated unigenes in SL221 cell biological pathways, KEGG pathway mapping was conducted. KEGG analysis can help identify the biological processes and/or phenotypic traits with which genes are associated [37]. Overall, 23630 annotated unigenes with significant matches were assigned to 283 known metabolic or signaling KEGG pathways (S2 Table). Among them, the most enriched pathways were “metabolic pathways”, “biosynthesis of secondary metabolites”, “ribosome”, “RNA transport”, “spliceosome”, and “protein processing in endoplasmic reticulum”. These annotations indicated potential directions for investigating specific processes, functions and signaling pathways in the infection of SpltNPV.

**Fig 2:**
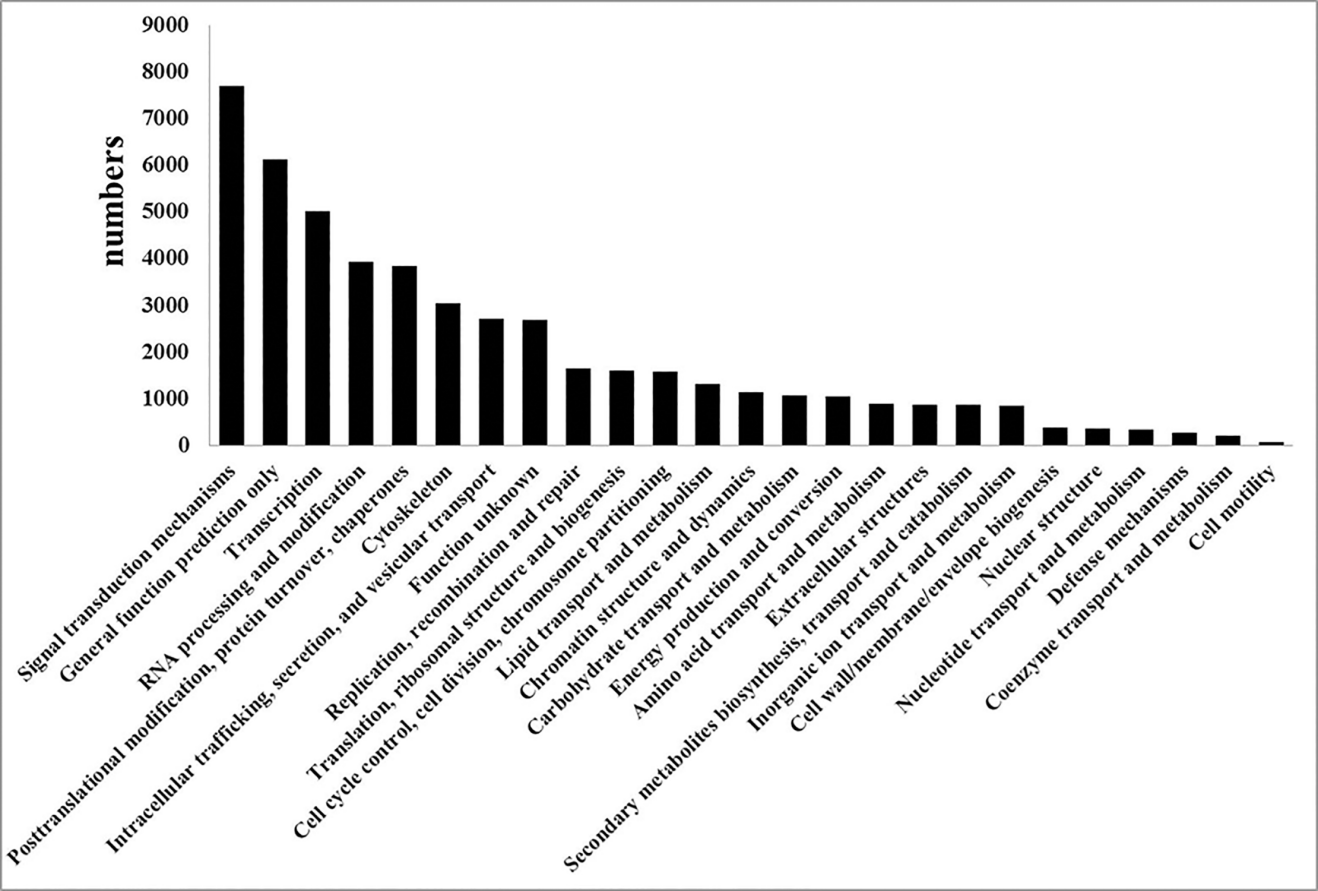
COG annotations of SL221 unigenes.

Unigenes were further searched against the COG database to analyze putative protein function. Overall, 16,141 unigenes were assigned to 83,872 COG classifications, which could be grouped into 25 categories (Fig 2). Among these 25 categories, the cluster for ‘signal transduction mechanisms’ represents the largest group (7,700 members) followed by ‘general function prediction’ (6,111 members) and ‘transcription’ (5,023 members), whereas the category ‘cell motility’ represented the smallest group (70 members). Several COG categories were probably associated with viral infection defense, including ‘defense mechanisms’ (270 members), ‘intracellular trafficking, secretion, and vesicular transport’ (2,709 members) and ‘cell cycle control, cell division, chromosome partitioning’ (1,577 members); 2,694 unigenes belonged to the ‘function unknown’ category.

### General Cellular Responses to SpltNPV Infection

**Fig 3:**
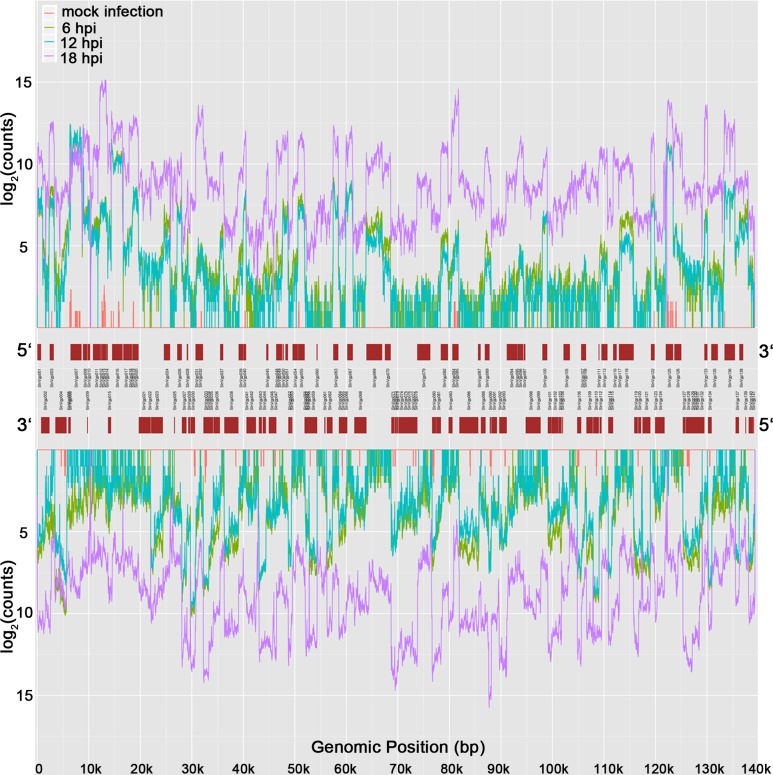
Unique ORF reads mapped to sense strand and anti-sense strand at 6, 12 and 18 hpi. 6 hpi: green line; 12 hpi: blue line; 18 hpi: purple line.

To determine the expression of SL221 cell unigenes and SpltNPV ORFs at different time points post infection, the screening RNA-seq reads from each time point of the three replicates groups were mapped to the 99,180 assembled unigenes (S3 Table). Finally, a total of 96,496 host unigenes together with SpltNPV 118 potential ORFs were at least 1 reads mapping to 3 out of 12 samples. Gene expression levels were determined as CPM values. As the results show, differential viral mRNAs began to increase remarkably after 6 hpi and represented 104 ORFs of SpltNPV by 18 hpi. By 18 hpi, approximately 2.7% of the measured total mRNA levels were from SpltNPV (S2 Fig). Of great interesting, as the reads from the samples in every four groups were respectively merged and strand-specifically aligned to SpltNPV genome, we found that each ORFs yielded different reads with peak value to the transcript from opposite strand (Fig 3). Especially for the opposite ORFs locating in same position of SpltNPV genome (such as slnVgp008 (sense strand) and slnVgp009 (anti-sense strand)), our strand-specific RNA-seq data reflected on these ORFs providing more accurate quantity for gene expression.

**Fig 4:**
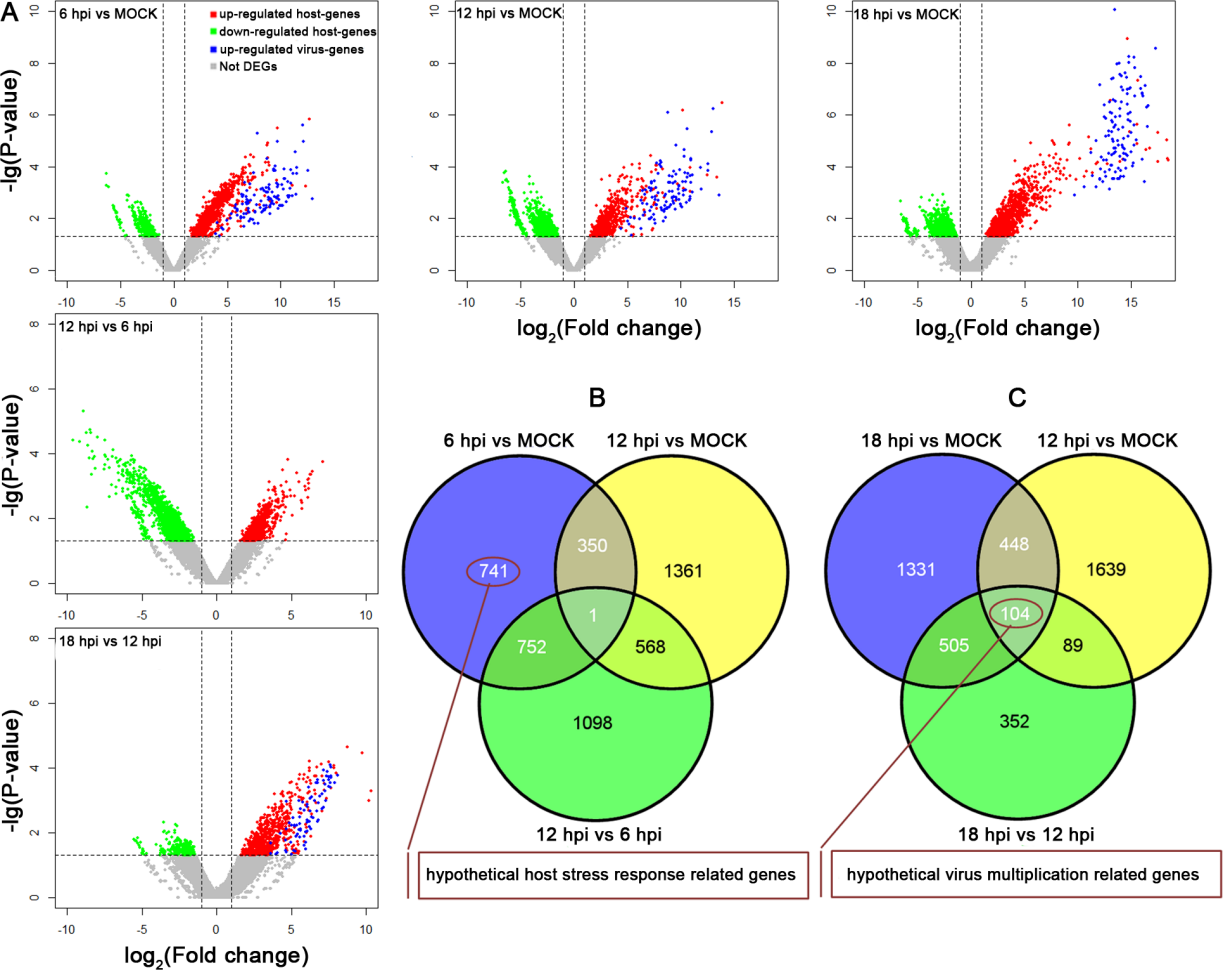
Volcano plots of up/downregulated and non-differentially expressed SL221 unigenes and viral genes throughout infection. A: Unigene expression levels between cells at 6/12/18 hpi and mock (horizontal axis), and unigene expression levels between adjacent infection time points (vertical axis). DEG: differentially expressed genes. B: Venn diagrams showing the protein overlap in biological replicates of cells at mock, 6 hpi and 12 hpi. C: Venn diagrams showing the protein overlap in biological replicates of cells at mock, 12 hpi and 18 hpi.

To analyze how viral infection caused changes in unigene expression, the expression level of each unigene at each time point post infection was compared with that of the same unigene at mock or at an adjacent infection time point (a parallel time point). As can be seen from Fig 4A (horizontal axis), compared with mock group, the proportion of upregulated viral genes are increased throughout the infection. The increasing trends were also shown in comparisons of adjacent infection times (Blue Spot, vertical axis), indicating that the viral gene expression level was increased step by step at the early infection stage. The number of SL221 DEG slightly increased throughout infection (from 1727 to 2270, horizontal axis). However, during comparisons of adjacent infection times, the number of SL221 DEGs increased at 12 hpi and climbed down at 18 hpi (vertical axis), with no differential expression, suggesting that SL221 cells responsed to viral infection stress before 12 hpi, and viral multiplication happened after 12 hpi.

The Venn diagram in Fig 4B and 4C shows the specific unigenes identified at different infection times. As can be seen in the Venn diagram (Fig 4B), 741 unigenes were observed to be specifically differential expressed at 6 hpi, considered to be hypothetical host stress response related genes. All these 741 DEGs were from SL221 cells, in which 62 unigenes were able to annotated as protein coding gene (S4 Table). Fig 4C showed that copmpared with 18 hpi and 12 hpi, 104 unigenes were observed to be specifically, considered to be hypothetical viral multiplication related genes, including 3 annotated SL221 genes and 81 viral genes (S5 Table).

### Enrichment Analysis

**Fig 5:**
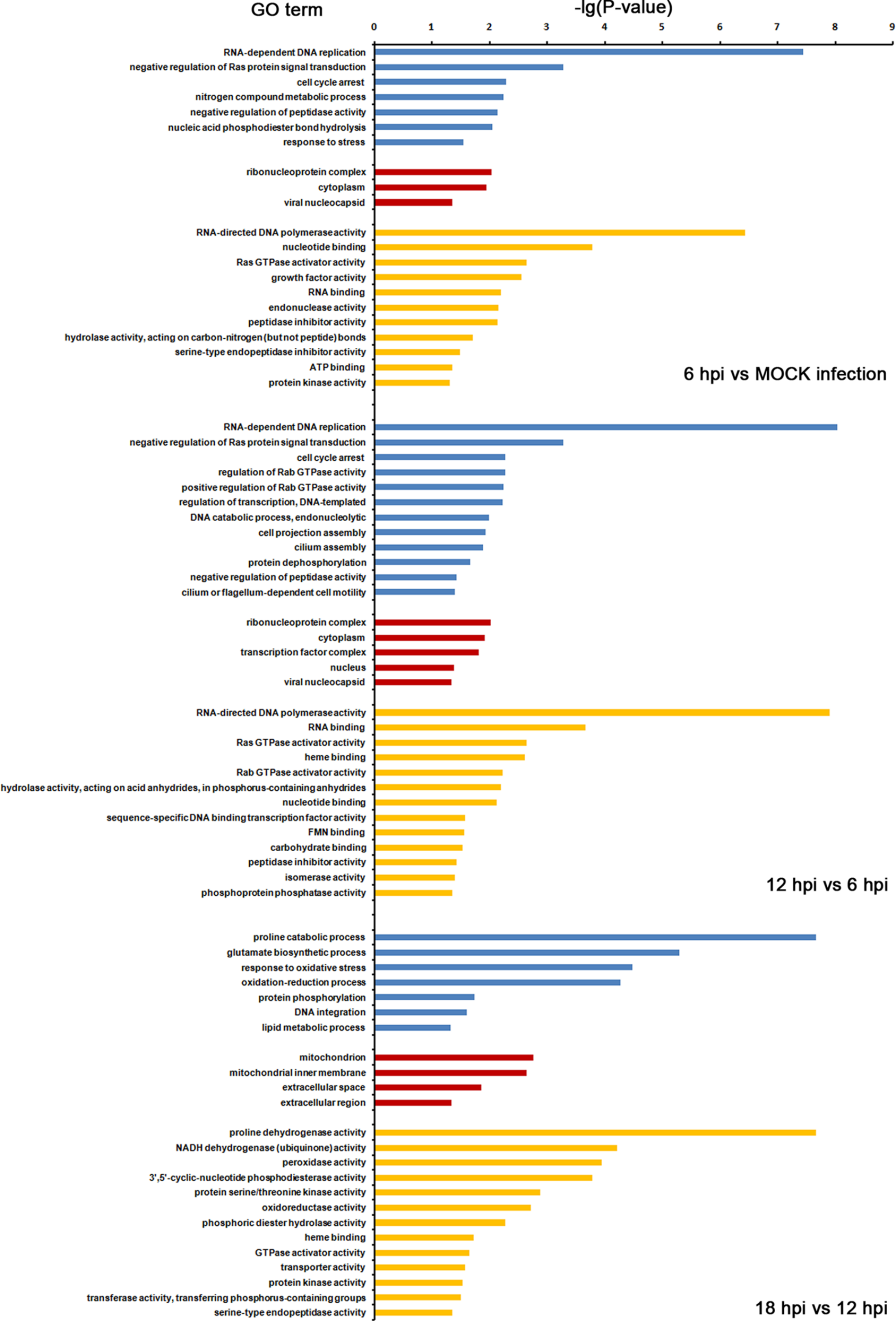
GO analysis of differentially expressed unigenes among different biological processes. GO analysis of differentially expressed SL221 transcripts was performed, and distributions of differentially expressed unigenes at 6 hpi (upper panel), 12 hpi (middle panel) and 18 hpi (lower panel) are shown. Blue represents biological process, Red represents cellular component and yellow represents molecular function.

To understand the functions and biological processes involved in infection, differentially expressed unigenes were enriched to GO terms, as shown in Fig 5. At 6 hpi, the most abundant GO terms were: RNA-dependent DNA replication, negative regulation of Ras protein signal transdution and cell cycle arrest (biological process); ribonucleoprotein complex, cytoplasm and viral nucleocaspid (cellular component); RNA-directed DNA polymerase activity and nucleotide binding (molecular function) (Fig 5 upper panel). At 12 hpi, the most abundant GO terms were: RNA-dependent DNA replication, negative regulation of Ras protein signal transdution and cell cycle arrest (biological process); ribonucleoprotein complex, cytoplasm and transcription factor complex (cellular component); RNA-directed DNA polymerase activity and RNA binding (molecular function) (Fig 5 middle panel). At 12 hpi, the most abundant GO terms were: proline catabolic process, glutamate biosynthetic process, response to oxidative stress and oxidative-reduction process (biological process); mitochodrion, mitocheondrial inner membrane and eextracellular space (cellular component); proline dehydrogenase activity, NADH dehydrogenase activity and peroxidase activity (molecular function) (Fig 5 lower panel).

Among the differentially expressed genes of the SL221 cells at 6 hpi, 2,181 unigenes were mapped to biological processes. Of the biological process-related genes, most upregulated unigenes between 6 hpi and mock-infected SL221 cells were enriched in RNA dependent DNA replication (16 unigenes), nucleic acid phosphodiester bond hydrolysis (5 unigenes) and response to stress (5 unigenes). In contrast, downregulated unigenes of this group were categorized as nitrogen compound metabolic process (2 unigenes) (Table 2). As for unigenes that showed >|2|-fold changes at 12 hpi (vs. 6 hpi, P<0.05), the upregulated genes were enriched in the following categories: regulation of transcription, DNA-templated (4 unigenes) and RNA dependent DNA replication (2 unigenes). The downregulated genes were mostly enriched in regulation of transcription, DNA-templated (28 unigenes), RNA dependent DNA replication (14 unigenes), protein dephosphorylation (6 unigenes), regulation of Rab GTPase activity (5 unigenes) and positive regulation of Rab GTPase activity (5 unigenes) (Table 3). Upregulated unigenes that showed >|2|-fold changes at 18 hpi (vs. 12 hpi, P<0.05) were mostly related to oxidation-reduction process (21 unigenes), protein phosphorylation (12 unigenes) and DNA integration (11 unigenes) (Table 4).

**Table 2:** GO term results of differential unigenes between 6 hpi and MOCK infected SL221 cells.

Gene Ontology term	Differential unigenes	UP	Down	P value	Unigenes annotated to term
RNA dependent DNA repli- cation	17 out of 1799 unigenes	16	1	3.58E-8	c58293_g1_i1_91272,c35908_g1_i1_36704,Contig28819_172152,Contig16463_158424,Contig13593_155235,Contig16220_158155,Contig2337_166098,c54799_g1_i1_85643,c57610_g1_i1_90233,Contig20411_162812,Contig16762_158756,Contig26598_169684,Contig15876_157771,Contig3303_176832,Contig13303_154914,Contig8365_184603, c51224_g5_i1_77942
negative regulation of Ras protein signal transduction	3 out of 8 unigenes	3	0	5.26E-4	Contig26704_169803,Contig8024_184225,Contig4814_180658
cell cycle arrest	3 out of 17 unigenes	3	0	5.19E-3	Contig17019_159042,c51547_g4_i2_79576,c51589_g1_i9_79816
nitrogen compound metabolic process	4 out of 30 unigenes	2	2	5.77E-3	Contig2164_164176,c51542_g1_i4_79541,c48818_g2_i1_67207,c62275_g1_i1_97796
negative regulation of peptidase activity	4 out of 32 unigenes	4	0	7.38E-3	c51734_g3_i4_80398,Contig16437_158395,c66607_g1_i1_104912,c51734_g3_i6_80400
nucleic acid phosphodiester bond hydrolysis	5 out of 495 unigenes	5	0	9.10E-3	Contig14251_155967,Contig16463_158424,Contig13593_155235,Contig32488_176229,c51729_g5_i4_80379,
response to stress	5 out of 64 unigenes	5	0	2.86E-2	c38827_g3_i1_43741,c51464_g1_i1_79097,Contig26768_169873,c68096_g1_i1_107155,c23040_g1_i1_18702

Unigenes > |2| fold, P **≤** 0.05 regulated between 6 hpi and MOCK infected SL221 cells based on Biological Process (p ≤ 0.05).

**Table 3:** GO term results of differential unigenes between 12 hpi and 6 hpi infected SL221 cells.

Gene Ontology term	Differential unigenes	UP	Down	P value	Unigenes annotated to term
RNA dependent DNA repli- cation	16 out of 1799 unigenes	2	14	9.30E-9	c38557_g6_i2_42924,c49644_g1_i1_71000,c58293_g1_i1_91272,Contig28819_172152,Contig16463_158424,Contig13593_155235,Contig16220_158155,Contig28877_172216,Contig13089_154675,Contig2337_166098,Contig16762_158756,Contig16058_157974,Contig26598_169684,Contig3303_176832,Contig28974_172324,Contig8365_184603
negative regulation of Ras protein signal transduction	3 out of 8 unigenes	0	3	5.38E-4	Contig26704_169803,Contig8024_184225,Contig4814_180658,
cell cycle arrest	3 out of 17 unigenes	0	3	5.30E-3	Contig17019_159042,c51547_g4_i2_79576,c51589_g1_i9_79816
regulation of Rab GTPase activity	6 out of 59 unigenes	1	5	5.38E-3	Contig2191_164476,c51761_g2_i3_80510,Contig5615_181548,Contig16613_158591,Contig28743_172068,Contig10354_151637
positive regulation of Rab GTPase activity	6 out of 60 unigenes	1	5	5.86E-3	Contig2191_164476,c51761_g2_i3_80510,Contig5615_181548,Contig16613_158591,Contig28743_172068,Contig10354_151637
regulation of transcription, DNA-templated	32 out of 655 unigenes	4	28	5.95E-3	c38493_g4_i1_42709,c53146_g1_i1_83022,c25954_g1_i1_22269,c51128_g1_i5_77465,c45243_g2_i1_55281,Contig15471_157322,Contig24389_167230,Contig7729_183896,c51486_g11_i9_79199,Contig27210_170366,Contig7432_183567,Contig27709_170919,c51497_g4_i1_79257,c51681_g2_i2_80215,Contig15130_156944,Contig15698_157573,Contig15712_157590,Contig8405_184648,Contig1694_158953,Contig16902_158912,c50379_g1_i10_73975,Contig16525_158493,c51497_g4_i3_79258,Contig8106_184316,Contig7593_183745,c51581_g3_i1_79778,c51508_g8_i5_79343,Contig15134_156948,Contig27040_170177,c51470_g3_i1_79122,Contig18410_160588,Contig30778_174329
DNA catabolic process, endonucleolytic	3 out of 21 unigenes	1	2	1.02E-2	c25102_g2_i1_21226,Contig23081_165778,Contig14022_155713
cell projection assembly	4 out of 36 unigenes	0	4	1.19E-2	Contig7906_184093,c51722_g2_i13_80362,c51722_g2_i4_80363,c51722_g2_i5_80364
cilium assembly	4 out of 37 unigenes	0	4	1.31E-2	Contig7906_184093,c51722_g2_i13_80362,c51722_g2_i4_80363,c51722_g2_i5_80364
protein dephosphorylation	7 out of 98 unigenes	1	6	1.70E-2	c12587_g1_i1_6954,Contig1599_157897,Contig15670_157543,Contig2005_162410,Contig8479_184729,Contig7920_184109,Contig2349_166231
negative regulation of peptidase activity	3 out of 32 unigenes	0	3	1.98E-2	c51734_g3_i4_80398,Contig16437_158395,c51734_g3_i6_80400
cilium or flagellum- dependent cell motility	4 out of 50 unigenes	0	4	2.04E-2	Contig7906_184093,c51722_g2_i13_80362,c51722_g2_i4_80363,c51722_g2_i5_80364

Unigenes > |2| fold, P ≤ 0.05 regulated between 12 hpi and 6 hpi SL221 cells based on Biological Process (p ≤ 0.05).

**Table 4:** GO term results of differential unigenes between 18 hpi and 12 hpi infected SL221 cells.

Gene Ontology term	Differential unigenes	UP	Down	P value	Unigenes annotated to term
proline catabolic process	4 out of 4 unigenes	4	0	2.13E-8	c35696_g1_i1_36358,Contig26995_170125,Contig6859_182929,Contig31817_175484
glutamate biosynthetic process	4 out of 18 unigenes	4	0	5.08E-6	c35696_g1_i1_36358,Contig26995_170125,Contig6859_182929,Contig31817_175484
response to oxidative stress	4 out of 28 unigenes	0	0	3.30E-5	c35734_g1_i1_36413,Contig32191_175900,Contig18652_160856,c8000_g1_i1_126246
oxidation-reduction process	21 out of 794 unigenes	21	0	5.34E-5	Contig6193_182190,c48593_g1_i2_66087,c68558_g1_i1_107901,Contig3241_176143,c35696_g1_i1_36358,Contig28797_172127,c35734_g1_i1_36413,Contig7248_183362,Contig26995_170125,c9686_g1_i1_147922,Contig21449_163964,Contig32191_175900,Contig18652_160856,Contig6859_182929,c40316_g1_i1_46330,c51885_g4_i1_80929,Contig15756_157638,c8000_g1_i1_126246,Contig31817_175484,Contig22668_165318,c66303_g1_i1_104462
protein phosphorylation	12 out of 610 unigenes	12	0	1.84E-2	Contig7300_183421,Contig25812_168812,Contig15460_157310,Contig3184_175509,c41876_g1_i1_48971,c33571_g1_i1_32954,Contig27411_170589,Contig12045_153516,c56886_g1_i1_89099,c52112_g1_i1_81366,c39086_g2_i2_44478,Contig29783_173223
DNA integration	11 out of 567 unigenes	11	0	2.52E-2	c6875_g1_i1_108221,c28524_g1_i1_25594,Contig31021_174601,c2659_g1_i1_23047,Contig8880_185175,Contig21089_163564,Contig1197_153431,Contig2309_165787,Contig3194_175620,c84896_g1_i1_132632,Contig29343_172735
lipid metabolic process	3 out of 108 unigenes	3	0	4.86E-2	Contig24321_167156,c38857_g1_i2_43845,c4214_g1_i1_49407

Unigenes >|2| fold, P ≤ 0.05 regulated between 18 hpi and 12 hpi SL221 cells based on Biological Process (p ≤ 0.05).

Among the differentially expressed genes of SL221 cells at 6 hpi (vs. mock, P<0.05), 532 unigenes were mapped to KEGG pathways. Most upregulated unigenes were enriched in biosynthesis of secondary metabolites (22 unigenes), endocytosis (14 unigenes), adrenergic signaling in cardiomyocytes (7 unigenes) and calcium signaling pathway (7 unigenes) (Table 5). As for unigenes that were changed by >|2|-fold at 12 hpi (vs. 6 hpi, P<0.05), the upregulated ones were enriched in the following pathways: biosynthesis of secondary metabolites (3 unigenes), FoxO signaling pathway (1 unigenes) and T cell receptor signaling pathway (1 unigenes). The downregulated genes were mostly enriched in biosynthesis of secondary metabolites (21 unigenes), HTLV-I infection (13 unigenes), cell cycle (10 unigenes), endocytosis (10 unigenes) and FoxO signaling pathway (10 unigenes) (Table 6). Upregulated unigenes that were changed by >2-fold at 18 hpi (vs. 12 hpi, P<0.05) were mostly related to oxidative phosphorylation (6 unigenes) and purine metabolism (5 unigenes) (Table 7).

**Table 5:** KEGG results of differential unigenes between 6 hpi and mock infected SL221 cells.

KEGG term	Differential unigenes	UP	Down	P value	Unigenes annotated to term
Endocytosis	14 out of 192 unigenes	14	0	9.92E-3	Contig2112_163599,Contig21305_163805,Contig2026_162643,c51531_g1_i2_79493,Contig28535_171837,c51577_g2_i5_79761,c51464_g1_i1_79097,c51582_g7_i4_79785,Contig27952_171189,Contig27387_170561,Contig8106_184316,Contig2054_162954,Contig8249_184474,c51903_g1_i2_81013
Adrenergic signaling in cardiomyocytes	7 out of 81 unigenes	0	0	1.91E-2	Contig29018_172374,Contig16302_158246,c88822_g1_i1_137536,Contig28675_171992,Contig28554_171858,Contig28059_171308,Contig27986_171226
Calcium signaling pathway	7 out of 90 unigenes	7	0	3.37E-2	c88822_g1_i1_137536,Contig27767_170983,Contig28675_171992,Contig28059_171308,Contig27986_171226,Contig2181_164365,Contig16675_158659
Phototransduction	3 out of 29 unigenes	3	0	4.13E-2	c88822_g1_i1_137536,Contig2054_162954,Contig7920_184109
Biosynthesis of secondary metabolites	22 out of 399 unigenes	22	0	4.22E-2	Contig7952_184144,Contig5013_180880,c51577_g2_i3_79759,Contig1996_162308,Contig1347_155098,Contig15472_157323,Contig16462_158423,c51899_g1_i2_80997,Contig2080_163243,Contig27913_171146,Contig28871_172210,Contig29057_172417,Contig15527_157384,Contig8526_184782,Contig24810_167699,Contig16533_158502,Contig7187_183294,c51631_g1_i7_80014,Contig27271_170433,c51542_g1_i4_79541,Contig19723_162046,Contig3438_178331

Unigenes > |2| fold, P ≤ 0.05 regulated between 6 hpi and mock infected SL221 cells based on KEGG database (p ≤ 0.05).

**Table 6:** KEGG results of differential unigenes between 12 hpi and 6 hpi SL221 cells.

KEGG term	Differential unigenes	UP	Down	P value	Unigenes annotated to term
FoxO signaling pathway	11 out of 120 unigenes	1	10	3.20E-3	c42144_g1_i1_49417,Contig29210_172588,Contig16483_158446,c51835_g1_i2_80782,Contig27040_170177,Contig16107_158029,Contig28554_171858,Contig26477_169550,Contig26768_169873,Contig8106_184316,Contig29297_172683
Biosynthesis of secondary metabolites	24 out of 399 unigenes	3	21	1.32E-2	c67474_g1_i1_106180,c5767_g1_i1_90331,c42144_g1_i1_49417,Contig7952_184144,Contig5013_180880,c51577_g2_i3_79759,Contig7254_183369,Contig1996_162308,Contig26763_169868,Contig16462_158423,c51899_g1_i2_80997,Contig27913_171146,Contig28871_172210,Contig29057_172417,Contig15527_157384,Contig8526_184782,Contig24810_167699,Contig16533_158502,Contig7187_183294,c51631_g1_i7_80014,Contig27271_170433,Contig19723_162046,Contig3438_178331,Contig26762_169867
Cell cycle	10 out of 138 unigenes	0	10	2.39E-2	Contig15608_157474,Contig28287_171561,c51835_g1_i2_80782,Contig7432_183567,Contig27040_170177,Contig28870_172209,Contig26477_169550,Contig26768_169873,Contig8106_184316,Contig1693_158942
HTLV-I infection	13 out of 204 unigenes	0	13	3.36E-2	Contig29210_172588,Contig1559_157453,c51835_g1_i2_80782,Contig7432_183567,Contig27040_170177,Contig27606_170805,Contig28870_172209,Contig28291_171566,Contig28059_171308,Contig22074_164659,Contig27510_170699,Contig8106_184316,Contig1693_158942
T cell receptor signaling pathway	8 out of 109 unigenes	1	7	3.50E-2	c12587_g1_i1_6954,Contig29210_172588,c51638_g2_i1_80049,Contig27210_170366,Contig28554_171858,Contig28870_172209,Contig27399_170574,Contig19723_162046,
Phototransduction	3 out of 29 unigenes	0	3	4.06E-2	c88822_g1_i1_137536,Contig2054_162954,Contig7920_184109
Morphine addiction	4 out of 44 unigenes	1	3	4.22E-2	c33670_g1_i1_33102,Contig14714_156481,Contig28059_171308,Contig2054_162954,
Endocytosis	12 out of 192 unigenes	2	10	4.50E-2	c43385_g1_i1_51573,c42949_g1_i1_50815,Contig21305_163805,Contig14714_156481,c51531_g1_i2_79493,Contig28535_171837,c51582_g7_i4_79785,Contig27952_171189,Contig8106_184316,Contig2054_162954,Contig8249_184474,c51903_g1_i2_81013
Phototransduction—fly	3 out of 30 unigenes	0	3	4.53E-2	Contig1859_160786,Contig14714_156481,c88822_g1_i1_137536
Epstein-Barr virus infection	4 out of 301 unigenes	0	4	4.61E-2	Contig29210_172588,Contig28554_171858,Contig28870_172209,Contig28291_171566,

Unigenes > |2| fold, P ≤ 0.05 regulated between 12 hpi and 6 hpi SL221 cells based on KEGG database (p ≤ 0.05).

**Table 7:** KEGG results of differential unigenes between 18 hpi and 12 hpi SL221 cells.

KEGG term	Differential unigenes	UP	Down	P value	Unigenes annotated to term
Oxidative phosphorylation	6 out of 134 unigenes	6	0	9.25E-4	c20949_g2_i1_16205,Contig28797_172127,c51885_g4_i1_80929,c4584_g1_i1_56644,c68558_g1_i1_107901,c47470_g1_i1_61523
Morphine addiction	3 out of 44 unigenes	3	0	2.00E-3	c39889_g2_i2_45656,Contig14723_156491,Contig1827_160431
Cardiac muscle contraction	3 out of 45 unigenes	3	0	2.17E-3	Contig28797_172127,c51885_g4_i1_80929,c47470_g1_i1_61523
Arginine and proline metabolism	3 out of 59 unigenes	3	0	5.74E-3	c35696_g1_i1_36358,Contig6859_182929,Contig31817_175484
Fc gamma R-mediated phagocytosis	3 out of 69 unigenes	3	0	9.91E-3	c9892_g1_i2_150279,c62969_g1_i1_98930,c52112_g1_i1_81366
Transcriptional misregulation in cancer	3 out of 101 unigenes	3	0	3.54E-2	c24616_g2_i1_20656,c5638_g1_i1_88291,c43470_g1_i2_51722
Purine metabolism	5 out of 225 unigenes	5	0	4.94E-2	Contig21449_163964,c39889_g2_i2_45656,c47332_g6_i12_61058,Contig1827_160431,c47332_g6_i10_61056

Unigenes > |2| fold, P ≤ 0.05 regulated between 18 hpi and 12 hpi SL221 cells based on KEGG database (p ≤ 0.05).

### Verification of Transcriptome Data by RT-qPCR

**Fig 6:**
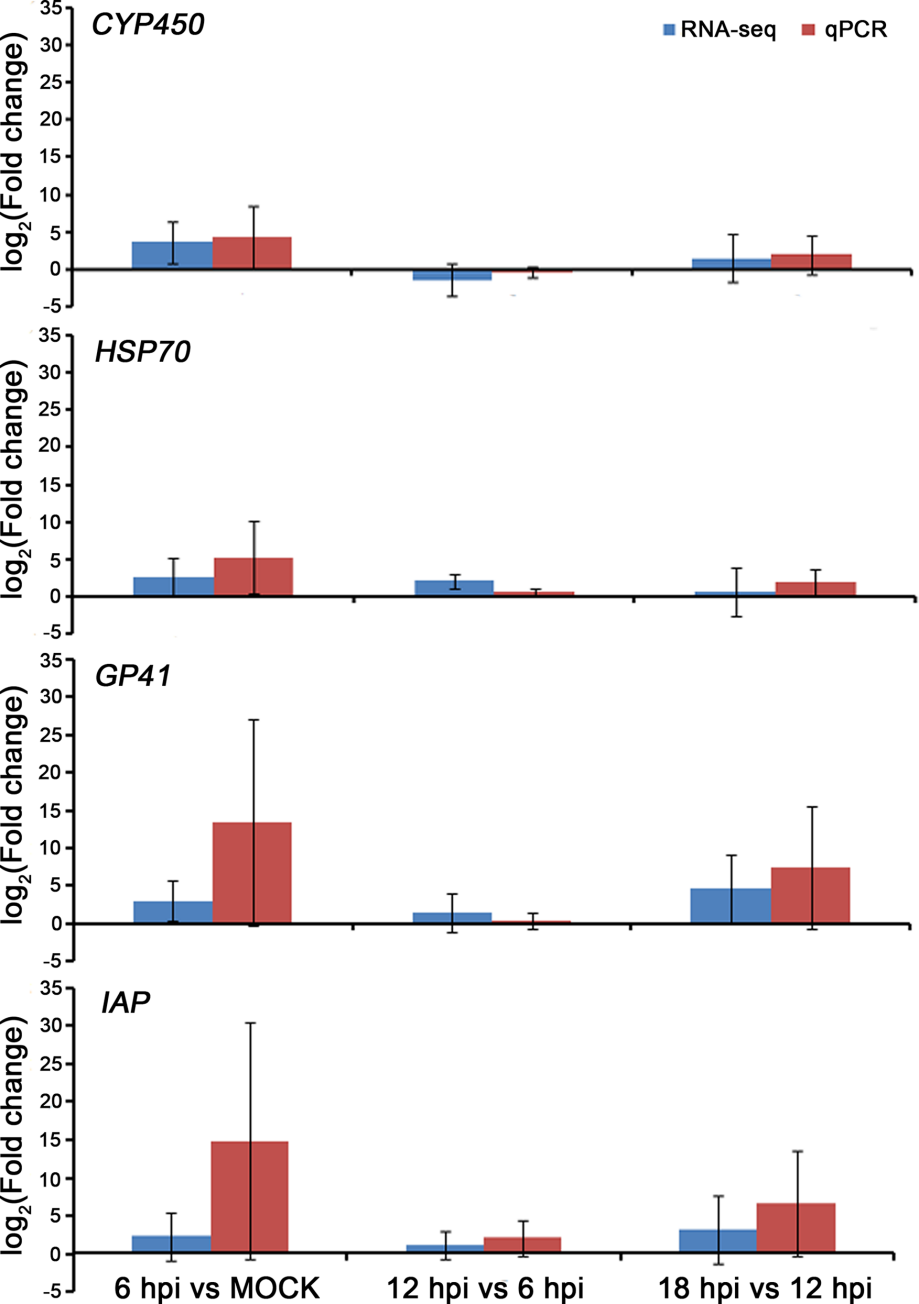
Validation of the RNA-seq results by quantitative real-time PCR (qRT-PCR). Two viral unigenes (GP41 and IAP) and 2 host cell unigenes (CYP450 and HSP70) with different fold changes were randomly selected for analysis using qRT-PCR, GAPDH was used for normalization.

To further evaluate our library of differentially expressed unigenes, 2 viral unigenes (IAP and GP41) and 2 host cell unigenes (CYP450 and HSP70) with different fold changes were randomly selected for analysis by qRT-PCR. These results are shown in Fig 6. The expression profiles all matched the results obtained using RNA-seq. Taken together, qRT-PCR analysis confirmed the changes detected by the mRNA sequencing analysis.

## Discussion

*S*. *litura*, which feeds on over 150 species of plants, is one of the most highly destructive agricultural pests in many countries [38]. One of the strategies used to control this insect is biological control, utilizing viruses to invade the insect host cells and destroy the function or inhibit the activity of host cells [39]. Changes in the transcriptome of SL221 cells during infection with SpltNPV directly reflect the impact of the virus on the cell activities and the cell responses that occur upon virus infection, which provides new insight into the biological control of these destructive agricultural pests.

In this study, we have characterized the transcriptome changes of *S*. *litura* cells infected by the SpltNPV at 0, 6, 12, 18 hpi. This represents the first investigation of transcriptome changes in SL221 cells as well as that of cell responses induced by infection of SpltNPV at the early stage. Moreover, Strand-specific sequencing can overcome the limitations of conventional Illumina RNA-Seq, such as lack of sufficient resolution to decode the complex eukaryote transcriptome without RNA polarity information. Therefore, more accurate gene expression evidence have shown that during the early infection stage, host cell cycle progression was arrested, and the cellular cytoskeleton was remodeling, preparing for the later propagation of virus. So changes in the host cell transcriptome at the early stage will show how host cell reponse to the virus infection stress.

For comparisons of differentially expressed unigenes resulting from viral infection, we used mock-infected cells as the controls, representing the state of uninfected cells. The results of GO and KEGG analyses showed that several of the differentially expressed genes were involved in multiple biological processes and pathways. At 6 hpi, most of the upregulated genes were involved in RNA dependent DNA replication, nucleic acid phosphodiester bond hydrolysis and response to stress. These genes were identified as those encoding pol-like protein, endonuclease-reverse transcriptase, reverse transcriptase, RNA-directed DNA polymerase, glutamate synthase, HSP70, HSP19.3, ribonuclease and lacunin. On the other hand, most of the upregulated genes were involved in nitrogen compound metabolic process, including genes encoding lacunin, KAL-1 and retrovirus-related pol polyprotein from transposon. During entry into the host cell, the virus can fuse its viral envelope with the plasma membrane. In addition, the virus also can enter the host cell via endocytosis, after which it escapes from the endosome by membrane fusion or lysis [40,41]. In fact, the entire process of viral infection is closely related to “transport” [42]. From retrograde transport (transport from the cell periphery to the center of the cell), to short range transport or anchoring in the vicinity of the nucleus and anterograde transport (transport from the cell center to the periphery), numerous membrane systems and their associated protein complexes were involved [41,43]. At a later stage (18 hpi), most of the upregulated genes are related to oxidation-reduction process, including unigenes that encode NADH-ubiquinone oxidoreductase chain 1 and 2, sluggish A, short-chain dehydrogenease/reductase, Xanthine dehydrogenase and superoxide dismutase.

In this study, cellular genes with different transcript levels across the 4 indicated infection times were grouped according to their potential physiological roles in SL221 cell growth and infection. Of the metabolic enzymes suggested to be regulated during culture growth, decarboxylase and hydroxylase are known to be involved in metabolism. Decarboxylase, sphingomyelin synthetase, adenosine deaminase related growth factor, GTP cyclohydrolase I and tyrosine hydroxylase were upregulated at 6 hpi, and at 18 hpi, the expression levels of DNA primase, DNA-directed RNA polymerase, thymidylate synthase isoform 1, pyridoxine 5'-phosphate oxidase and DNA polymerase were significantly upregulated and classified under ‘metabolic pathway’, emphasizing the dependency of SpltNPV replication on cell metabolism. In contrast, the expression levels of fatty acid synthase, GJ24310, lipin-3 and glucosylceramidase were significantly decreased at 6 hpi, and those of cytochrome CYP6AE47, hexokinase, glutathione S-transferase, DOPA decarboxylase, chondroitin synthase, UDP-glycosyltransferase and glutamine synthetase were also significantly decreased. Decreased anabolic fluxes through metabolic pathway were previously reported [44]. The increased replication of viral DNA was consistent with the results of previous studies [27].

During viral infection, the production of stress response-related genes in the host cells was increased in the very early infection stage (6 hpi). At 6 hpi, 1844 unigenes were signifcantly differentially expressed. These gene may be associated with host cell response to viral infection at the early stage through facilitating viral replication and assembly, and they may cause the virus to be recognized by the host immune system and thus cause the infected cells to be eliminated [45]. Numerous genes participate in this host cell stress response. Among all these genes, Heat shock proteins (HSPs) represent a class of stress response genes that are associated with protein folding or unfolding [46,47]. The results from this study showed that the transcript levels of HSP70 unigenes were significantly increased at 6 hpi. At 12 hpi and 18 hpi, several HSP unigenes were upregulated, including HSP20.7, HSP20.8, HSP15.0, HSP22, HSP20.4, HSP40, HSP19.7, HSP27.2, HSP23.7 and HSP90. The upregulation of these HSPs suggests an association between the disturbance of endoplasmic reticulum (ER) homeostasis and SpltNPV infection. Cytochrome P450, which participate in metabolism of signal molecules, was significantly increased at 6 hpi, followed by a reduction at 12 hpi and significant reduction at 18 hpi [48], suggesting that cytochrome P450 may be adaptation to virus resistance. In these unigenes, 741 out of 1844 unigenes were considered to be hypothetical host stress response related genes due to specifically differential expressed at 6 hpi. Diverse enzymes, receptor and ligand protein genes involved stress response with SpltNPV infection. On the other hand, we found that later in the early infection stage (12–18 hpi), 104 unigenes, including 23 of SL221 cell ones and 81 of viral ones, were continuously significantly changing, considered to be hypothetical viral multiplication related genes. Of the 81 viral unigenes, tetraspanin was remarkable. The tetraspanin superfamily, first recognized in 1990, has now grown to about 20 known members. Studies have shown that Tetraspanin proteins play important roles in cellular penetration, invasion, and fusion events and define a novel type of membrane microdomain [49]. The tetraspanin superfamily can inertact many other signaling molecules and play parts in activation, adhesion and cell differentiation. They act as “molecular facilitators”, bring together specific cell-surface proteins and allow them function more efficiently. Apoptosis is an important defense mechanism in many organisms. Through direct or indirect inhibition of apoptosis, viruses can evade monitoring by the host immune system [50]. During the release of the viral particles in the late stage of infection, virus-encoded proteins can also induce host-cell apoptosis. How host cells sense viral infection and initiate apoptosis is therefore critical to the outcome of an infection [51].The transcript level of the SpltNPV apoptosis inhibitor *spltNPV-iap* was dramatically increased at 6 hpi, decreased to its lowest level at 12 hpi, and increased again at 18 hpi. Since the discovery that baculoviruses can manipulate the apoptotic response of their insect hosts, much progress has been made in determining how these viral genes, IAPs, and their cellular counterparts actually function at the biochemical level to inhibit apoptosis [52]. During infection, the control of host cell translation and replication is an integral part of the life cycle of a virus. Previous studies have shown that viral IAP can impede cell death in insect cells and prevent cell death in mammalian cells [53,54]. Due to IAP will be important tools in defining the pathways involved in apoptosis during infection, we evaluated the expression of *iap* in SpltNPV infected SL221 cells. qRT-PCR results confirmed that upon SpltNPV infection, the expression of *SpltNPV-iap* was increased at 6 hpi, followed by a significant decrease at 12 hpi. At 18 hpi, the expression was again significantly increased.

## Conclusions

This transcriptome study offers new insights and provides potential research targets for a better understanding of *S*. *litura* cellular responses to SpltNPV-G2 infection. Our data highlight how viral infection relates to host defensive responses at early stage. In a broader sense, this work and future studies can provide more targets for the use of viral infection in biological insect control and/or other biomedical and biotechnological applications involving baculoviruses.

## Supporting Information

S1 Fig Insect species that have high sequence homology with the annotated unigenes. (TIF)

S2 Fig SpltNPV ORF changes at various time points post-infection. (TIF)

S1 Table Unigene annotations (protein coding). (XLSX)

S2 Table Unigene pathway. (XLS)

S3 Table Unigene annotations. (XLSX)

S4 Table Descriptions of hypothetical host stress response related protein coding genes. (DOCX)

S5 Table Descriptions of hypothetical virus multiplication related protein coding genes. (DOCX)
