# Supplementary figures and images for: Transcriptome Analysis of the SL221 Cells at the Early Stage during Spodoptera litura Nucleopolyhedrovirus Infection
Source: PLoS One. 2016 Feb 3;11(2):e0147873. doi: 10.1371/journal.pone.0147873 (PMC4739724; doi:10.1371/journal.pone.0147873)

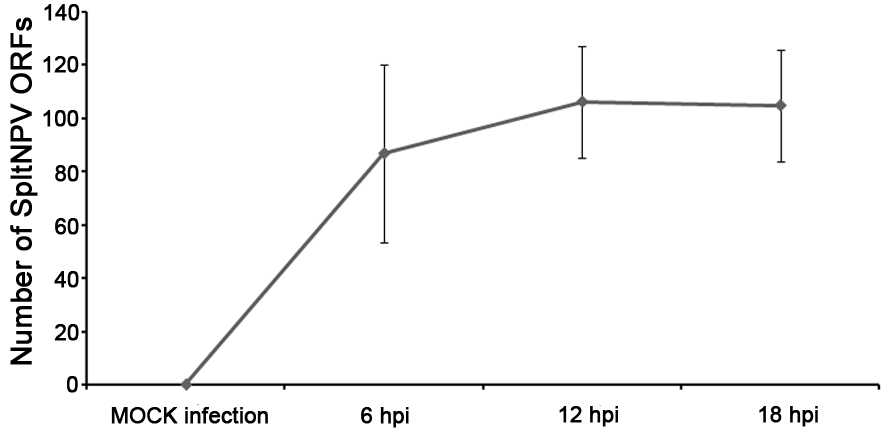

Supplement: S1 Fig — (TIF) [file pone.0147873.s001.tif]

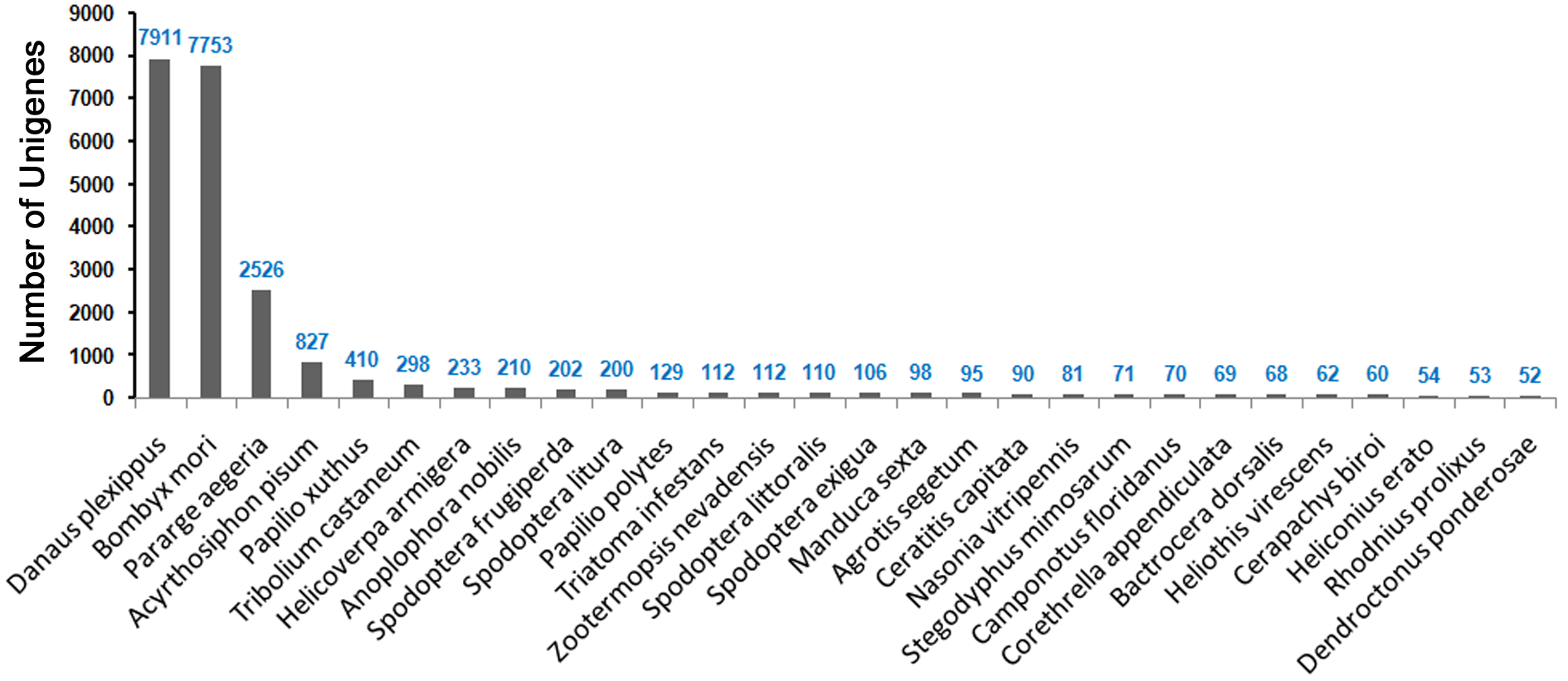

Supplement: S2 Fig — (TIF) [file pone.0147873.s002.tif]
