# Supplementary material for: Transcriptome Analysis of the SL221 Cells at the Early Stage during Spodoptera litura Nucleopolyhedrovirus Infection
Source: PLoS One. 2016 Feb 3;11(2):e0147873. doi: 10.1371/journal.pone.0147873 (PMC4739724; doi:10.1371/journal.pone.0147873)
Supplement: S4 Table — (DOCX) [file pone.0147873.s006.docx]

**Table S4.**

**Description of hypothetical host stress response related protein coding genes.**

| Unigene_ID | Log_2_(FC) | PValue | Gene_name | protein_description | blast_species |
| --- | --- | --- | --- | --- | --- |
| c18863_g1_i1_14040 | -1.99356 | 0.040822 | PGBD4 | PiggyBac transposable element-derived protein 4 | Hydra vulgaris |
| c19936_g1_i1_15166 | -2.28435 | 0.043133 | POL3 | Retrovirus-related Pol polyprotein | Anoplophora nobilis. |
| c20355_g1_i2_15601 | -2.05392 | 0.029387 | mod(mdg4) | Mod(Mdg4)-heS00531 | Bombyx mori |
| c24513_g1_i2_20525 | -2.4777 | 0.034978 | CYP15C1 | Cytochrome P450 15C1 | Bombyx mori |
| c30713_g1_i1_28702 | -2.07802 | 0.039772 | OR | Putative odorant receptor OR43 | Cydia pomonella |
| c44073_g1_i3_52814 | -1.94245 | 0.039858 |  | Methenyltetrahydrofolate synthetase domain- containing protein | Pararge aegeria |
| c45566_g3_i3_55994 | -2.73425 | 0.041183 |  | Solute carrier family 35 member F5 | Pararge aegeria |
| c46279_g1_i3_57730 | -1.84389 | 0.049248 | KGM_11294 | AMP dependent coa ligase | Danaus plexippus |
| c48818_g2_i1_67207 | -2.33953 | 0.043598 | nit1 | Nitrilase | Heliothis subflexa |
| c51224_g5_i1_77942 | -3.31283 | 0.018739 |  | Putative retrovirus-related pol polyprotein from transposon | Corethrella appendiculata. |
| c51520_g9_i2_79413 | 2.638044 | 0.029654 |  | RNA polymerase-associated protein Rtf1 | Pararge aegeria |
| c51542_g1_i4_79541 | 2.0756 | 0.030415 | KGM_05167 | Glutamate synthase | Danaus plexippus |
| c51618_g5_i1_79948 | 2.213513 | 0.043837 |  | EG:BACR37P7.3 | Pararge aegeria |
| c51652_g1_i1_80107 | 2.117504 | 0.026486 | KGM_11062 | Putative retinoid X receptor | Danaus plexippus |
| c51713_g8_i2_80323 | 3.710822 | 0.003608 | FKRLMTSLSM | Annexin | Heliothis virescens |
| c51720_g3_i2_80350 | 2.886402 | 0.029567 | LOC692843 | Defective in cullin neddylation protein | Bombyx mori |
| c51739_g4_i10_80429 | 1.790932 | 0.037716 |  | Anoctamin | Bombyx mori |
| c51782_g2_i9_80598 | 2.036955 | 0.043831 | KGM_08640 | Glucosylceramidase | Danaus plexippus |
| c52345_g1_i1_81725 | 2.887097 | 0.036189 | KGM_09659 | Putative maintenance of killer 16 protein | Danaus plexippus |
| c52483_g1_i1_81958 | -2.23619 | 0.041589 | Dvir\GJ24310 | GJ24310 | Drosophila virilis |
| c54415_g1_i1_84998 | 2.006971 | 0.04704 | Dpse\GA27597 | GA27597 | Drosophila pseudoobscura |
| c57070_g1_i1_89391 | 2.891121 | 0.034113 | polyprotein | Polyprotein | Drosophila melanogaster |
| c62275_g1_i1_97796 | -2.00539 | 0.035304 | nit1 | Nitrilase | Heliothis virescens |
| c90275_g1_i1_139302 | 1.791519 | 0.044654 |  | Coronin | Bombyx mori |
| c98069_g1_i1_149431 | 2.611801 | 0.016949 | polyprotein | Polyprotein | Drosophila melanogaster |
| Contig13303_154914 | 2.345745 | 0.027621 | GFP_L5_0210 | Retroelement polyprotein | Glyptapanteles flavicoxis. |
| Contig14797_156572 | 1.952985 | 0.037649 |  | Yellow-f4 | Papilio xuthus |
| Contig14864_156647 | 2.032371 | 0.043824 |  | Alanyl-tRNA synthetase | Pararge aegeria |
| Contig15027_156829 | 2.765647 | 0.014477 | KGM_15128 | Putative netrin 1a | Danaus plexippus |
| Contig15629_157497 | 2.208428 | 0.028925 |  | Rho guanine nucleotide exchange factor 12 | Pararge aegeria |
| Contig15876_157771 | 2.399978 | 0.028615 | ELSDPYKMEC | RNA-directed DNA polymerase from mobile element jockey | Pararge aegeria |
| Contig16089_158008 | 3.172784 | 0.008566 | KGM_04571 | Putative F-box/WD-repeat protein pof1 | Danaus plexippus |
| Contig16448_158407 | 2.62072 | 0.012701 |  | Pastrel | Pararge aegeria |
| Contig16529_158497 | 3.532069 | 0.004208 | KGM_15130 | Uridine kinase | Danaus plexippus |
| Contig1702_159043 | 3.140511 | 0.025209 |  | Roadkill | Pararge aegeria |
| Contig1720_159243 | 2.131792 | 0.036228 | KGM_21655 | Putative cytoplasmic dynein light chain | Danaus plexippus |
| Contig1764_159731 | 2.806968 | 0.013972 | slo | Slowpoke | Drosophila buzzatii |
| Contig1840_160576 | 1.678521 | 0.047729 | slimb | Slimb | Danaus plexippus |
| Contig1869_160897 | 2.36431 | 0.035619 | ATF | Fatty alcohol acetyltransferase | Agrotis segetum |
| Contig1928_161553 | 2.102599 | 0.049312 | KGM_13160 | Paternally expressed 3 | Danaus plexippus |
| Contig1936_161642 | 2.89874 | 0.011086 |  | Held out wings | Pararge aegeria |
| Contig2033_162721 | 2.891694 | 0.018256 |  | Simila to CG1648 | Papilio polytes |
| Contig2080_163243 | 2.245195 | 0.027356 |  | S-adenosylmethionine synthase | Bombyx mori |
| Contig2112_163599 | 2.573625 | 0.015393 |  | Putative tyrosine kinase negative regulator cbl | Triatoma infestans |
| Contig2185_164409 | 3.421188 | 0.01065 | KGM_10033 | Putative GTP-binding protein | Danaus plexippus |
| Contig2381_166587 | 1.775181 | 0.028396 |  | Anoctamin | Bombyx mori |
| Contig24059_166864 | 2.116575 | 0.039792 |  | Putative feline leukemia virus subgroup c receptor-related protein2 | Corethrella appendiculata. |
| Contig2510_168021 | 3.128281 | 0.023581 | KGM_06769 | Putative coiled-coil domain containing 13 | Danaus plexippus |
| Contig26030_169055 | 2.053478 | 0.040111 |  | Guanylate cyclase | Manduca sexta |
| Contig27118_170263 | 1.962759 | 0.031519 |  | Tyrosine-protein kinase receptor | Bombyx mori |
| Contig27387_170561 | 2.893011 | 0.021196 |  | Chromatin modifying protein 1b | Bombyx mori |
| Contig27714_170925 | 2.343917 | 0.034208 | KGM_16411 | Amino acid transporter | Danaus plexippus |
| Contig27733_170946 | 2.617265 | 0.010407 | SLKNSLRHET | Diapause hormone receptor | Helicoverpa zea |
| Contig27815_171037 | 1.981329 | 0.044769 |  | Annexin | Helicoverpa armigera |
| Contig27870_171098 | 2.99821 | 0.015734 | KGM_13482 | Ras small monomeric GTPase Rab6 | Danaus plexippus |
| Contig27986_171226 | 2.247233 | 0.02234 | IDIQMGMLNH | Ryanodien receptor | Spodoptera exigua |
| Contig28206_171472 | 2.593407 | 0.021699 | KGM_20963 | Putative paramyosin | Danaus plexippus |
| Contig28376_171660 | 2.523254 | 0.027602 | KGM_10468 | Putative glucuronyltransferase I | Danaus plexippus |
| Contig28955_172303 | 2.376902 | 0.008307 | KGM_04632 | DNA-directed RNA polymerase | Danaus plexippus |
| Contig4046_179805 | 2.712661 | 0.037281 |  | Antennal esterase CXE6 | Spodoptera littoralis |
| Contig509_180964 | 1.868179 | 0.048653 | KGM_20502 | Malic enzyme | Danaus plexippus |
| Contig7693_183856 | 2.052204 | 0.036132 | PLIN | PLIN | Bombyx mori |
