# Supplementary material for: Transcriptome Analysis of the SL221 Cells at the Early Stage during Spodoptera litura Nucleopolyhedrovirus Infection
Source: PLoS One. 2016 Feb 3;11(2):e0147873. doi: 10.1371/journal.pone.0147873 (PMC4739724; doi:10.1371/journal.pone.0147873)
Supplement: S5 Table — (DOCX) [file pone.0147873.s007.docx]

**Table S5.**

**Description of hypothetical virus multiplication related protein coding genes.**

| unigene | Log_2_(FC) | PValue | Gene_name | protein_description | blast_species |
| --- | --- | --- | --- | --- | --- |
| c20949_g2_i1_16205 | 17.49367 | 6.63E-05 | ATP6 | ATP synthase subunit | Spodoptera litura |
| c41876_g1_i1_48971 | 14.63439 | 1.12E-09 |  | major occlusion protein,protein kinase | Paramecium tetraurelia. |
| c42141_g1_i1_49409 | 5.348415 | 0.000129 |  | Tetraspanin | Bombyx mori |
| SlnVgp001 | 17.27088 | 2.77E-09 |  | major occlusion protein | Spodoptera litura nucleopolyhedrovirus |
| SlnVgp002 | 14.21154 | 3.01E-06 | pp78/81 | essential structural protein pp78/81 | Spodoptera litura nucleopolyhedrovirus |
| SlnVgp008 | 13.94378 | 5.93E-07 | IE-0 | IE-0 | Spodoptera litura nucleopolyhedrovirus |
| SlnVgp010 | 13.2163 | 4.19E-08 |  | dUTP pyrophosphatase | Spodoptera litura nucleopolyhedrovirus |
| SlnVgp011 | 13.29529 | 3.92E-08 |  | hypothetical protein | Spodoptera litura nucleopolyhedrovirus |
| SlnVgp012 | 14.80534 | 9.61E-09 |  | occlusion-derived virus envelope protein ODV-E18 peptide | Spodoptera litura nucleopolyhedrovirus |
| SlnVgp013 | 14.09438 | 8.26E-08 |  | occlusion-derived virus envelope proteinODV-EC27 | Spodoptera litura nucleopolyhedrovirus |
| SlnVgp014 | 13.70262 | 1.11E-08 |  | hypothetical protein | Spodoptera litura nucleopolyhedrovirus |
| SlnVgp017 | 14.92619 | 1.75E-06 |  | occlusion-derived virus envelope proteinODV-E56 | Spodoptera litura nucleopolyhedrovirus |
| SlnVgp018 | 15.91713 | 3.38E-08 |  | hypothetical protein | Spodoptera litura nucleopolyhedrovirus |
| SlnVgp019 | 13.44117 | 8.90E-11 | P10 | P10 protein | Spodoptera litura nucleopolyhedrovirus |
| SlnVgp020 | 15.268 | 6.05E-09 |  | hypothetical protein | Spodoptera litura nucleopolyhedrovirus |
| SlnVgp021 | 11.261 | 3.00E-05 | P74 | P74 protein | Spodoptera litura nucleopolyhedrovirus |
| SlnVgp024 | 14.74417 | 6.82E-06 |  | hypothetical protein | Spodoptera litura nucleopolyhedrovirus |
| SlnVgp029 | 13.19422 | 4.47E-06 | LEF-6 | LEF-6 | Spodoptera litura nucleopolyhedrovirus |
| SlnVgp031 | 12.49511 | 1.20E-06 |  | hypothetical protein | Spodoptera litura nucleopolyhedrovirus |
| SlnVgp032 | 15.34387 | 1.56E-08 |  | ubiquitin GP37 fusion protein | Spodoptera litura nucleopolyhedrovirus |
| SlnVgp034 | 14.49175 | 3.37E-05 | LEF-11 | LEF-11 | Spodoptera litura nucleopolyhedrovirus |
| SlnVgp035 | 12.9925 | 0.000137 |  | hypothetical protein | Spodoptera litura nucleopolyhedrovirus |
| SlnVgp037 | 14.41595 | 5.73E-05 | LEF-12 | LEF-12 | Spodoptera litura nucleopolyhedrovirus |
| SlnVgp038 | 14.31723 | 5.34E-05 | LEF-8 | LEF-8 | Spodoptera litura nucleopolyhedrovirus |
| SlnVgp039 | 13.95749 | 4.01E-05 | bJDP | bJDP | Spodoptera litura nucleopolyhedrovirus |
| SlnVgp042 | 13.32544 | 2.66E-07 | chitinase | chitinase | Spodoptera litura nucleopolyhedrovirus |
| SlnVgp044 | 12.92486 | 6.48E-06 |  | hypothetical protein | Spodoptera litura nucleopolyhedrovirus |
| SlnVgp045 | 12.92721 | 3.06E-05 |  | hypothetical protein | Spodoptera litura nucleopolyhedrovirus |
| SlnVgp046 | 12.06224 | 7.18E-08 |  | hypothetical protein | Spodoptera litura nucleopolyhedrovirus |
| SlnVgp047 | 12.46994 | 4.09E-07 |  | hypothetical protein | Spodoptera litura nucleopolyhedrovirus |
| SlnVgp052 | 16.14238 | 1.84E-07 |  | hypothetical protein | Spodoptera litura nucleopolyhedrovirus |
| SlnVgp053 | 12.60781 | 6.72E-06 |  | hypothetical protein | Spodoptera litura nucleopolyhedrovirus |
| SlnVgp054 | 13.84094 | 3.84E-06 |  | cathepsin-like cysteine proteinase | Spodoptera litura nucleopolyhedrovirus |
| SlnVgp057 | 13.8789 | 1.04E-08 | FP | few polyhedra protein | Spodoptera litura nucleopolyhedrovirus |
| SlnVgp058 | 13.07761 | 7.99E-07 |  | hypothetical protein | Spodoptera litura nucleopolyhedrovirus |
| SlnVgp064 | 14.37855 | 2.02E-05 | IAP | apoptosis inhibitor | Spodoptera litura nucleopolyhedrovirus |
| SlnVgp065 | 12.22183 | 4.42E-05 |  | hypothetical protein | Spodoptera litura nucleopolyhedrovirus |
| SlnVgp066 | 13.39853 | 1.96E-05 |  | hypothetical protein | Spodoptera litura nucleopolyhedrovirus |
| SlnVgp068 | 13.23168 | 1.94E-06 |  | hypothetical protein | Spodoptera litura nucleopolyhedrovirus |
| SlnVgp071 | 14.42531 | 2.44E-08 |  | hypothetical protein | Spodoptera litura nucleopolyhedrovirus |
| SlnVgp072 | 14.56632 | 3.65E-08 |  | hypothetical protein | Spodoptera litura nucleopolyhedrovirus |
| SlnVgp073 | 14.07463 | 3.17E-08 |  | hypothetical protein | Spodoptera litura nucleopolyhedrovirus |
| SlnVgp074 | 14.85155 | 9.53E-08 | VLF-1 | VLF-1 | Spodoptera litura nucleopolyhedrovirus |
| SlnVgp075 | 14.89176 | 9.30E-08 |  | hypothetical protein | Spodoptera litura nucleopolyhedrovirus |
| SlnVgp076 | 14.23776 | 1.13E-07 | GP41 | glycoprotein GP41 | Spodoptera litura nucleopolyhedrovirus |
| SlnVgp077 | 13.96845 | 2.77E-08 |  | hypothetical protein | Spodoptera litura nucleopolyhedrovirus |
| SlnVgp078 | 13.19828 | 8.89E-07 |  | telokin-like protein-20 | Spodoptera litura nucleopolyhedrovirus |
| SlnVgp079 | 14.98833 | 4.83E-06 | VP91 | VP91 | Spodoptera litura nucleopolyhedrovirus |
| SlnVgp080 | 13.3388 | 1.06E-05 | CG30 | zinc finger protein CG30 | Spodoptera litura nucleopolyhedrovirus |
| SlnVgp081 | 14.6684 | 1.58E-07 | VP39 | VP39 capsid | Spodoptera litura nucleopolyhedrovirus |
| SlnVgp082 | 14.53644 | 0.00012 | LEF-4 | LEF-4 | Spodoptera litura nucleopolyhedrovirus |
| SlnVgp083 | 14.67431 | 3.84E-07 |  | hypothetical protein | Spodoptera litura nucleopolyhedrovirus |
| SlnVgp084 | 12.6594 | 1.41E-06 |  | hypothetical protein | Spodoptera litura nucleopolyhedrovirus |
| SlnVgp085 | 13.50119 | 2.96E-07 |  | occlusion-derived virus envelope proteinODV-E25 | Spodoptera litura nucleopolyhedrovirus |
| SlnVgp088 | 12.96684 | 3.14E-07 |  | hypothetical protein 38K | Spodoptera litura nucleopolyhedrovirus |
| SlnVgp089 | 15.28644 | 1.52E-05 | LEF-5 | LEF-5 | Spodoptera litura nucleopolyhedrovirus |
| SlnVgp090 | 14.79536 | 5.61E-09 | P6.9 | P6.9 | Spodoptera litura nucleopolyhedrovirus |
| SlnVgp091 | 15.33487 | 1.87E-07 |  | hypothetical protein | Spodoptera litura nucleopolyhedrovirus |
| SlnVgp092 | 16.07378 | 1.60E-07 |  | hypothetical protein | Spodoptera litura nucleopolyhedrovirus |
| SlnVgp093 | 14.89898 | 8.00E-07 |  | hypothetical protein | Spodoptera litura nucleopolyhedrovirus |
| SlnVgp094 | 14.70176 | 1.58E-06 | VP80 | capsid-associated protein VP80 | Spodoptera litura nucleopolyhedrovirus |
| SlnVgp096 | 15.87606 | 9.67E-08 |  | hypothetical protein | Spodoptera litura nucleopolyhedrovirus |
| SlnVgp098 | 13.20453 | 1.68E-06 | ODV-E66 | occlusion-derived virus envelope protein ODV-E66 | Spodoptera litura nucleopolyhedrovirus |
| SlnVgp099 | 13.52433 | 2.73E-05 | P13 | P13 protein | Spodoptera litura nucleopolyhedrovirus |
| SlnVgp101 | 16.46031 | 5.09E-07 |  | hypothetical protein | Spodoptera litura nucleopolyhedrovirus |
| SlnVgp102 | 16.55264 | 2.90E-06 |  | hypothetical protein | Spodoptera litura nucleopolyhedrovirus |
| SlnVgp107 | 13.50885 | 1.24E-06 |  | hypothetical protein | Spodoptera litura nucleopolyhedrovirus |
| SlnVgp108 | 13.36389 | 3.10E-06 |  | hypothetical protein | Spodoptera litura nucleopolyhedrovirus |
| SlnVgp109 | 16.04797 | 5.26E-05 | Alk-exo | alkaline exonuclease | Spodoptera litura nucleopolyhedrovirus |
| SlnVgp112 | 14.3311 | 8.41E-06 |  | hypothetical protein | Spodoptera litura nucleopolyhedrovirus |
| SlnVgp113 | 15.45354 | 9.62E-06 |  | hypothetical protein | Spodoptera litura nucleopolyhedrovirus |
| SlnVgp115 | 13.93728 | 1.14E-06 |  | hypothetical protein | Spodoptera litura nucleopolyhedrovirus |
| SlnVgp116 | 14.73629 | 9.37E-07 | P24 | P24 capsid | Spodoptera litura nucleopolyhedrovirus |
| SlnVgp117 | 12.08971 | 0.000289 |  | hypothetical protein | Spodoptera litura nucleopolyhedrovirus |
| SlnVgp119 | 16.06184 | 1.43E-07 |  | hypothetical protein | Spodoptera litura nucleopolyhedrovirus |
| SlnVgp124 | 14.22976 | 1.85E-05 |  | hypothetical protein | Spodoptera litura nucleopolyhedrovirus |
| SlnVgp126 | 13.51015 | 1.92E-05 |  | hypothetical protein | Spodoptera litura nucleopolyhedrovirus |
| SlnVgp127 | 12.89797 | 4.39E-06 |  | hypothetical protein | Spodoptera litura nucleopolyhedrovirus |
| SlnVgp128 | 13.5316 | 2.55E-06 |  | 38.7 kDa protein | Spodoptera litura nucleopolyhedrovirus |
| SlnVgp129 | 16.6298 | 4.54E-07 | LEF-1 | LEF-1 | Spodoptera litura nucleopolyhedrovirus |
| SlnVgp130 | 14.66985 | 1.01E-07 |  | hypothetical protein | Spodoptera litura nucleopolyhedrovirus |
| SlnVgp131 | 15.24433 | 2.57E-06 |  | hypothetical protein | Spodoptera litura nucleopolyhedrovirus |
| SlnVgp132 | 14.11563 | 3.21E-07 |  | calyx protein | Spodoptera litura nucleopolyhedrovirus |
| SlnVgp133 | 14.66093 | 3.70E-05 | pkip | pkip | Spodoptera litura nucleopolyhedrovirus |
